# Life cycle assessment of recycling metallised food packaging plastics using mechanical, thermal and chemical processes

**DOI:** 10.1016/j.heliyon.2024.e36547

**Published:** 2024-08-19

**Authors:** Samy Yousef, Inga Stasiulaitiene

**Affiliations:** a Department of Production Engineering, Faculty of Mechanical Engineering and Design, Kaunas University of Technology, LT-51424, Kaunas, Lithuania; b Department of Environmental Technology, Kaunas University of Technology, Radvilenu str. 19, LT-50254, Kaunas, Lithuania

**Keywords:** Metallised food packaging plastics, Pyrolysis, Catalytic pyrolysis, Mechanical, Chemical, Life cycle analysis

## Abstract

Single treatment of metallised food packaging plastics waste (MFPW) has shown disappointing results with recycling rate <20 % due to its complex structure consisting of 10 % aluminium (Al) and 90 % mixed plastic films made of PE, PP, PS, PET, etc. Besides, it is generating many emissions and residues that must be landfilled making it difficult to integrate them into the circular economy. Therefore, a multi-stage recycling (MSR) approach has recently been developed using several sequential mechanical, thermal and chemical processes to recover energy and Al from MFPW with additional revenue for recycling plant operators. The thermal treatment helps to decompose the plastic fraction into wax or oil, gaseous, and solid residue (SR) composed of Al and coal, while the mechanical process can be used as a pre-treatment of MFPW feedstock and SR. Finally, the chemical treatment (leaching and functionalization) can be used to extract Al from SR and to refine coal into carbon microparticles (CPs), respectively. In order to investigate the environmental performance of the proposed MSR system, this research was developed. The investigation was performed using SimaPro life cycle analysis (LCA) tool according to ISO 14040/44 Standards and the impact assessment method is ReCiPe 2016. Five different scenarios were proposed in the constructed LCA layout, namely, conversion of MFPW to a) wax and gas (pyrolysis), b) wax, gas, and aluminium chloride (AlCl₃) (pyrolysis and leaching), c) wax, gas, AlCl₃, and CPs (pyrolysis, leaching, and functionalization), and d) oil, gas, AlCl₃, and CPs (catalytic pyrolysis, leaching, and functionalization). Besides, the oil produced from catalytic pyrolysis is used for generation of electricity (scenario e). The results showed that wax and gas recovery scenario (a) has better environmental potential and environmental benefits compared to incineration practice. The results did not change much after extraction of Al and CPs (scenario b, c), with a few increasing by 2–4% in the total score. While a lot of environmental burdens from upgrading and utilization (Scenario d, e) were recorded, reaching 79 % due to the huge amount of the catalyst was used. Thus, MSR systems have bigger environmental benefits, however, the chemical and catalytic processes still need to be further improved to reduce the effect of terrestrial acidification.

## 1 Introduction

Plastic packaging is the largest plastic consumption market in Europe with an estimated utilization rate of 40 % of its total plastic production, which has reached nearly 62 million tons recently [1]. Plastic food packaging (PFP) accounts for the bulk of this consumption and due to its short service life (few days that can be increased to a few weeks), huge amounts of PFP waste are produced daily [2,3]. Metallised food packaging plastics waste (MFPW) represents a major and complex fraction in PFP because it contains aluminium (Al: ∼10 %) and mixture of various plastic films (like PE, PP, PS, PET, etc.) joined together by the mechanical and chemical bonds in need of special management [4,5]. Irresponsible disposal of MFPW has a significant negative influence on environment and oceans and several harmful impacts on marine life and humans [6,7]. In order to reduce these negative effects, some countries have banned the use of plastic packaging and replaced it with other biodegradable materials like wood and paper [8,9]. However, these kinds of packaging cannot be used in case of some wet products for a long time. Besides, their raw materials are not always available in many countries which makes plastic materials an indispensable partner in this sector [10,11]. Therefore, recycling and energy recovery is the ideal way to manage this waste and to convert it to energy or raw materials, what helps in conservation of resources by displacing the use of virgin materials and involving them in the circular economy [12]. However, this needs more technical and legislative requirements, therefore, less than 43 % of waste are incinerated in thermal plants and the remaining fraction is disposed in landfill [13].

Recently, some flexibility has emerged in this framework, as some technologies have been adapted to that proposal, including mechanical, chemical and thermal treatments [14]. Shredding mechanical treatment can help with sorting, washing, milling, extrusion processes to reprocess plastic waste that can be used to produce mixed-plastics granulate as a raw material [15]. However, these granules have many shortcomings, such as low mechanical properties and poor compatibility as the feedstock is a mixed waste plastic consisting of many polymer fractions, hence leading to production of secondary raw plastic with potential applications of lower quality and economic value, which makes the choice of potential application arbitrary [[16], [17], [18]]. Also, mechanical option is mainly applicable and highly recommended for sorted mono-material not mixed fractions [19]. This means that this method is inefficient for recycling of multi layers and heterogeneous plastic waste like MFPW and for sorting of incoming waste plastic to improve its quality. Therefore, attention has been turned to chemical recycling approach converting plastic waste into monomers, higher hydrocarbons, etc., or separating them into their original layers [20,21]. However, this practice has a lot of environmental burdens and consumes a lot of chemicals, which can affect quality of the valuable products extracted [22,23]. Thermal treatments are another type of advanced recycling technologies that have been developed recently to convert plastic waste into basic chemicals and volatile compounds and gases [[24], [25], [26]]. Pyrolysis is one such technology that has recently reached the stage of pilot scale, where MFPW can be decomposed to paraffin wax [27,28]. Meanwhile, catalytic pyrolysis can be used to upgrade wax into oil and to increase the yield of flammable gases in syngas product [29,30]. However, solid residues (SR), including coal and Al, are usually disposed of in landfills, causing more environmental burdens. Therefore, SR was exposed to various types of mechanical and chemical processes (e.g. milling, leaching, and functionalization) to extract Al and to reprocess coal fraction into carbon microparticles (CPs), what contributed to improvement of its economic performance, as well [31,32]. Although a multi-stage recycling (MSR) approach combining mechanical, thermal, and chemical processes succeeded in recovering all components of MFPW (in form of Al, CPs, and energy products) with high recovering rate and economic performance, the environmental performance is still missing. Life cycle assessment (LCA) is a standardized global approach used as a valuable checking tool to support the environmental studies and management of waste and to evaluate improvements or compare several viable treatment technologies in terms of environmental footprint [33,34]. Several studies were developed to study the LCA of management plastic waste using different treatments in form of single stage treatment or multi-stages, then compared with landfill and incineration scenarios [[35], [36], [37]]. The studies showed different conclusions due to changing the perspective of the studies (waste, product quality, replacement ratio with virgin materials, utilization, etc.). However, in general, pyrolysis and chemical recycling techniques manifested a significant reduction in the impact of climate change compared to incineration and mechanical practices. Also, the results showed that the analysis is very sensitive to the suggested LCA layout, including the input (materials and energy), output items (electricity recovered), emissions, geographical location, etc. [[37], [38], [39]].

Unfortunately, there is little information in the literature about LCA for MFPW and the study by Ahmed et al.(2020) is considered as distinguished on this topic [6]. In this study, the authors studied the environmental impact of converting MFPW to oil via pyrolysis treatment, while the obtained gases were used to synthesize carbon nanotubes (CNTs) using chemical vapor deposition (CVD). However, the study was focused on oil and CNTs products and neglected the procedures of Al extraction. Besides, the procedures of pre-treatment and effect of nitrogen gas used in the reaction were not taken into consideration. Also, the coal fraction was disposed in landfill, what affected the accuracy of the calculated impacts. In addition, the study was conducted on a specific type of mixed polymer waste, what makes it difficult to apply it at industrial scale. Besides, more sorting processes are required, where any changes in the composition of feedstock affect the generated syngas and structure of the synthesized CNTs and its yield [40]. All these restrictions can be treated using MSR approach as listed above, as these solutions were developed based on a mixture of commercial products and treated under similar processing conditions as those used on an industrial scale, what helps to increase transparency and accuracy of the results. In order to disclose the environmental performance MSR of MFPW, this research was developed. The LCA was performed based on five perspectives: wax production (pyrolysis), Al extraction (chemical leaching), Al-free char particles (ACP) reprocessing into CPs (chemical functionalization), wax upgrading for oil production (catalytic pyrolysis), and utilization of oil in electricity production. The LCA layouts and analysis were performed based on the experimental results previously published by our group [4,27,[29], [30], [31], [32],^41^]. Finally, the developed MSR strategy for recycling of MFPW was compared to pyrolysis process as an industrial alternative that follows the concept of a single stage treatment.

## 2 Design of the research and methodology

The current research structure consists of five main phases, including a) characterization and determination of the composition of MFPW feedstock, b) definition of the conditions and boundaries of each process used in the developed MSR approach to convert MFPW into energy, Al, and CPs products, c) building of the LCA scheme of the proposed MSR approach in the form of successive processes, including their inputs and outputs, and d) analysis of the environmental performance of the entire the developed MSR strategy for treating MFPW. All of these phases and their assumptions are explained in detail in the following sections to estimate the environmental impacts.

### 2.1 Characterization of feedstock

**Fig 1:**
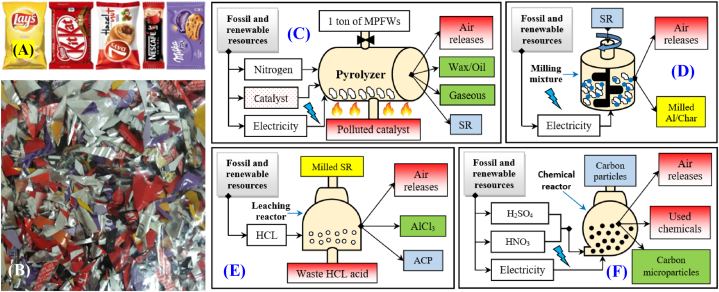
A, B) images of the selected MFPW feedstock and their shredded fraction, respectively, and system boundaries of C) pyrolysis or catalytic pyrolysis process, D) milling pre-treatment, E) leaching treatment, and F) functionalization process.

The LCA was carried out on feedstock of mixture of MFPW (potato chips, chocolate, bakery products, coffee and biscuits) distributed equally and composed of 91 wt% of polymer fraction and 9 wt% of Al fraction (Fig. (1A)). These food products are characterized by their high demand rate among all generations (children, young, and old) in the EU region. This feedstock is composed of different polymer layers made of PET, LDPE, EVA, etc. and all compositions are listed [4,41]. Also, the feedstock is very rich in carbon (82.24 %), hydrogen (14.07 %), and volatile matter (90.652 %) based on Elemental and proximate analysis [27].

### 2.2 Treatment of MFPW using MSR approach and their boundaries

The selected MFPW raw materials were processed by the proposed MSR approach in five phases: a) shredding pre-treatment of MFPW, b, c) pyrolysis or catalytic pyrolysis treatment, d) milling pre-treatment of SR, d) Al leaching from SR, and e) coal refining using functionalization process. All conditions and system boundaries of each phase are illustrated in Fig. (1B-F) and explained in the following sections.

#### 2.2.1 Shredding pre-treatment of MFPW

The system boundary of the entire MSR strategy begins with the pre-treatment of the collected MFPW using shredding process by a chopping machine to decrease size of MFPW (less than 10 mm as shown in Fig. (1B)) and its crystallinity, as well [17], which contributed to increase of the surface contact with the heating ambient, hence, helping to make heating exchange faster and to achieve full decomposition in the next thermal conversion treatments [42]. The pre-treatment was performed at room temperature and 500 rpm [43]. Since the moisture content in the shredded MFPW is very low (only 0.04 %) [4], no drying was required.

#### 2.2.2 Pyrolysis treatment of MFPW

This stage was employed to convert MFPW into wax and gaseous energy products. The boundaries of this phase were built based on the optimal operating conditions obtained experimentally from our previous study [27]. The pyrolysis experiments were carried out in a small pyrolysis plant with capacity of 250 g, at 20 °C/min, nitrogen flow rate 5 L/min, pyrolysis temperature 600 °C, for residence time 60 min. At these conditions, the polymer fraction was decomposed to gas (62.1 wt%), wax (19.4 wt%), and SR (18.5 wt%), as indicated in (Fig. (1C)). All of these variables were set as real operating conditions on an industrial scale, as well. Also, it was assumed that generation efficiency and products distribution from this treatment and the following processes were the same as the results from the experiments under the specified conditions. Finally, and based on the suggested layout, wax was the only product derived from pyrolysis treatment as a final product, while the obtained gaseous by-product can be recycled back to the pyrolysis chamber to maintain the pyrolysis plant operating based on the findings of other studies [44]. Pyrolysis gas was considered an energy product in this study with an estimated heating value of 4221 MJ/FU (for pyrolysis) and 4422 MJ/FU (for catalytic pyrolysis) as shown in **Table [S1]** in the Supplementary Information section. Thus, SR fraction can be considered as a semi product composed mainly of two fractions with different compositions, sizes, and morphologies: Al flakes and coal need further processing to separate these components and turn them into final products.

#### 2.2.3 Catalytic pyrolysis treatment of MFPW

FPW catalytic pyrolysis treatment was used to upgrade wax and to produce high-quality pyrolysis oil and gas rich in flammable gases. The layout of this stage was designed based on the results obtained from the catalytic pyrolysis experiments performed with a ZSM-5 zeolite catalyst to MFPW ratio (w/w) of 50 % using the same pyrolysis plant as mentioned above under the same conversion conditions [29,30]. By the end of the treatment, MFPW was decomposed into gas (66.09 wt%), oil (20.98 wt%), and SR (12.93 wt%) (Fig. (1C)). The pyrolysis oil fuel produced from such feedstock and process is classified as non-acidic and non-corrosive and it has a chemical structure and composition similar to fossil fuels with a high calorific value making it an alternative source for low sulphur diesel production [36]. Also, during the process, hydrocarbons compounds in non-condensable formulated gaseous products decomposed under the effect of catalytic process into CH4-rich fuel gas [30]. Finally, gas and oil were in the form of finished products, while SR was a semi-finished product that needed further refining, as mentioned before.

#### 2.2.4 Milling pre-treatment of SR

The milling process was used as another pre-treatment to decrease the size of SR fraction (especially Al flakes) obtained from pyrolysis or catalytic pyrolysis treatments and to make it in the uniform shape and distribution, thus preparing SR powder with high degree of fineness (Fig. (1D)) [43]. This shape helps to increase the surface contact between SR particles and chemical media during leaching and functionalization processes (in the following processing steps) and to accelerate the reaction and digest Al [45]. This process was performed using Ball milling at 20 Hz for 1 h and the layout was constructed without any output product [27], as the SR powder is still a semi-finished product.

#### 2.2.5 Leaching treatment of SR

The milled SR powder was exposed to chemical leaching treatment to dissolve Al fraction, then extracting it in the form of aluminium chloride (AlCl₃). The treatment was carried out using 3 Molar concentration (3 M)-Nitric acid (HNO_3_) with solvent-to-powder ratio = 3 (w/w) under the effect of soundwave at 50 °C for 3 h. [32], thus separating AlCl₃ against coal powder after filtration and drying process. The boundaries of this stage were constructed based on these conditions and AlCl₃ as a finished product, while coal powder still needs to be further refined. It is not considered a final product in the proposed layout, as described in (Fig. (1E)).

#### 2.2.6 Functionalization treatment of SR

Chemical functionalization treatment was used to refine ACP and to convert it into CPs by removing any contaminants and impurities from it [31]. The treatment was performed using ultrasonic process at 150 °C for 30 min using sulphuric acid (H_2_SO_4_): HNO_3_ = 3:1 without any additional washing process to avoid generation of more waste by the end of treatment and subsequently, drying was applied. CPs were the final product of this process and the boundaries are shown in (Fig. (1F)).

### 2.3 Environmental impact from the life cycle perspective

LCA of the suggested MSR recycling system of MFPW was studied based on ISO 14040:2006, ISO 14044:2006 international environmental standards using SimaPro software based on cradle-to-gate approach by defining a goal and scope, inventory data collection for each process, and environmental impact assessment for full strategy in the form of several items [46]. In the suggested model, MFPW feedstock loaded zero burden and started accumulating burden from the phase gate approach.

#### 2.3.1 Goal and scope

The goal of this study is to compare the environmental impact of different pathways for treatment of MFPW using an integrated system (MSR) composed of different thermal, mechanical, and chemical processes without sending any residue to an inert waste sanitary landfill. The scope was focused on conducting a LCA system covering the entire treatments and their recovered main products (wax or oil, and gas) and co-products (AlCl₃ and CPs). Also, the utilization of the upgraded wax (oil) in electricity generation and its substitution in energy sector was involved in the study scope. Such studies can help to develop more sustainable and environmental solutions, while their policy helps decision makers and investors to apply it at the industrial level [47], especially as the status of treatments (e.g., pyrolysis, etc.) is still under development within TRL 6 [36], what means that 5–7 years are needed for future developments and application at industrial scale (assuming, this could happen in 2030) and this is in line with the EU vision and circular economy [48]. The study assumes that the considered MSR system is applicable in the European countries. Therefore, the geographical background of the present LCA research and recycling process was set and adopted in Europe.

#### 2.3.2 Functional unit and study constrains

The functional unit (FU) used in the present research was defined as recycling of 1 ton of MFPW comprising several polymer layers of PET, LDPE, EVA, etc. (90 %) and Al (10 %). This composition was defined by SimaPro software and it was assumed that all types of polymer films were evenly distributed in the feedstock. Since the scope of study is focused on the recycling technical part only, the collection, transportation from households, and other activities and sorting procedures prior to MFPW arrival at the recycling plant were excluded from the suggested LCA layout. Besides, plastic feedstocks are usually transported for use as feed to any type of processing and waste management system in place. In addition, until now there has been no clear industrial vision for collection and sorting of MFPW which makes the estimation of the costs of collection and sorting an inaccurate estimate and further studies are needed for that purpose.

#### 2.3.3 System boundaries of the entire MSR system

**Fig 2:**
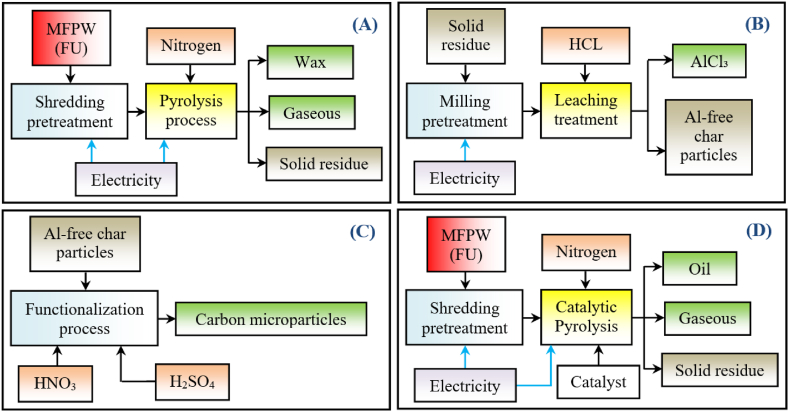
Constructed system boundaries of A) pyrolysis of MFPW, B) Al leaching from the solid residue fraction, C) functionalization of char particles, and D) catalytic pyrolysis of MFPW.

**Fig 3:**
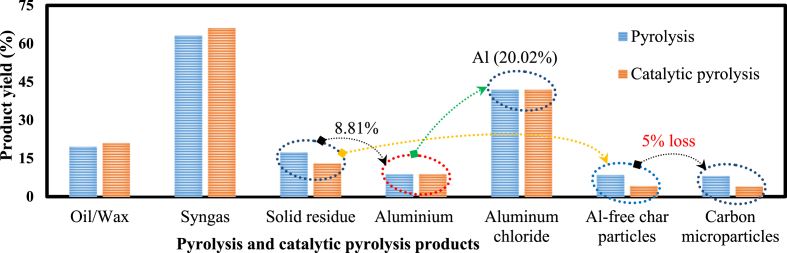
Yields of the obtained products using pyrolysis and catalytic pyrolysis.

The MFPW was processed in five stages and the boundaries of each process are shown in Fig. (1). In order to evaluate the environmental impacts of the entire proposed MSR recycling strategy of MFPW, all these processes were connected together, then the LCA layout of each scenario (pyrolysis of MFPW, Al leaching, functionalization of char particles, and catalytic pyrolysis of MFPW) was re-built again as a set of operation units and the constructed layouts are shown in Fig. (2A-D). As shown, the boundaries of MSR system were built based on the inputs (energy and materials), outputs (emissions, waste and products recovered), and utilization. Also, the displacement effect between the recycling and production processes was neglected to avoid generating more associated emissions [36]. Finally, the yields of the obtained products in the form of semi- or final product are summarized in Fig. (3). In case of pyrolysis, the yield was estimated for wax (19.6 %), syngas (63 %), and SR (17.4 %), while in case of catalytic pyrolysis, it was estimated for oil (21 %), syngas (66 %), and SR (13 %). In both cases, Al and ACP were estimated at 8.8 % and 3.12 %, respectively with 5 % less during preparation of Al salt and purification of CPs.

#### 2.3.4 Scenario descriptions

Five different scenarios were suggested to select the optimal option based on the higher environmental performance. The experimental results of the scenarios were tested in the laboratory [27,30,31]. As mentioned above, MFPW is a complex waste that needs several subsequent processes to be fully processed without sending any residue to a landfill. Unfortunately, studies have focused on extracting the Al fraction using a leaching process or converting the polymeric fraction into oil using pyrolysis, which leads to the production of more waste that must be disposed of in a landfill or is valuable through other processes (such as), as recent studies have proven [27,30,31]. However, studying the environmental impact of each process in the entire life cycle and its contribution individually will not be accurate because in this case there will be no residue from these different stages as they will be treated as input raw materials for the next process, which will affect the accuracy of the calculation. Also, the pyrolysis pathway and catalytic pyrolysis (main treatments) are not the same because the manufactured products are not the same. Accordingly, different scenarios are very necessary. The perspectives of the suggested scenarios were proposed based on the recovered products (wax, oil, AlCl₃, CPs) and their utilization. The products recovery was estimated on the assumption that these products can be replaced and substituted by AlCl₃ and CPs raw materials and energy products. In the utilizations perspective, the pyrolysis oil was combusted as an input feedstock to generate electricity (with conversion efficiency estimated at 34 %) to substitute the electricity derived from conventional fossil fuels, including coal and natural gas, which represent the main sources of electricity production in Europe, with an estimated contribution of 11 % (coal), and natural gas (34 %) [49]. The utilization perspective was studied for potential utilizations of the recovered oil (upgraded wax) in electricity generation sector, where this item was excluded from the system boundaries of other recovered products’ perspective. Finally, the conversion efficiency of transforming pyrolysis oil to electricity was estimated at 34 % based on the reported results in the literature. This means that the estimation of the produced electricity per functional unit pyrolysis oil is 3.2 kWh [50]. The heat required for electricity generation process was supplied from the recovered pyrolysis oil and its inherent calorific values are shown in **Table [S1]**. The harvested heat from syngas combustion was recycled back into the conversion plant based on similar assumptions in the literature [44]. Finally, the hypothesis of these scenarios was proposed based on the following items: wax production, Al extraction, CPs synthesis, wax upgrading to oil fuel, and utilization of oil fuel in electricity production, as described in Table [1].

**Table [1]:** The hypothesis of the suggested scenarios under the present study.

Code	Scenario	Scenario perspective	Process
S1	Scenario (A)	Conversion of MFPW to wax and gaseous	Pyrolysis
S2	Scenario (B)	Conversion of MFPW to wax, gaseous, and AlCl₃	Pyrolysis and leaching
S3	Scenario (C)	Conversion of MFPW to wax, gaseous, AlCl₃, and CPs	Pyrolysis, leaching, and functionalization
S4	Scenario (D)	Conversion of MFPW to oil, gaseous, AlCl₃, and CPs	Catalytic pyrolysis, leaching, and functionalization
S5	Scenario (E)	Conversion of MFPW to oil, gaseous, AlCl₃, CPs, and electricity	Catalytic pyrolysis, leaching, functionalization, and utilizations

#### 2.3.5 Benchmarks practices

In order to investigate the technical and ecological potential of MSR strategy to convert MFPW to energy and raw materials according to the suggested perspectives, scenario (A) was assessed and compared to the traditional practice recovery of energy from plastic waste via incineration, which was used as benchmark for that propose. Afterwards, scenario (A) was used as a baseline to evaluate the performance of other scenarios (B-E). It was assumed that the recovered pyrolysis wax and oil displaced the “energy recovery”, “diesel production, and low-sulphur” datasets, respectively [36]. Meanwhile, the recovered AlCl₃ and CPs displaced the ‘treatment of aluminium scrap, post-consumer, prepared for recycling, at remelter’ and ‘carbon microparticles production, low-quality’ datasets, respectively. The electricity supply for the used treatments and the electricity production scenario was build based on the Energy market authority of the EU and the EU electricity production from fossil fuel.

#### 2.3.6 Life cycle inventory

The proposed LCA model for MSR system was build based on the experimental findings previously published by our group [4,27,[29], [30], [31], [32],^41^]. Other missing data were collected from the literature and other sources based on a methodological and consistent perspective. The collected data from lab-scale experiments and other different sources were extended to 1 ton of MFPW (FU) based on a mass balance principle and the inventory data for mechanical, thermal, and chemical treatments, and their sources are described below.

##### 2.3.6.1 Life cycle inventory of mechanical treatment

Two main mechanical treatments were involved in the suggested layout, including shredding and milling process. Liang et al. (2022) estimated the energy required for shredding of 1 kg of plastic mixture by 54.45 kJ/kg (15.125 kWh/ton) [43], while the grinding needed to refine the graphite was calculated by Naimi et al. (2018) and adopted in the range 0.011–0.057 kWh/kg [51]. Due to the high roughness of SR, the maximum value (0.057 kWh/kg) was used as energy consumed during milling process. Based on the obtained yield of SR, the energy consumption was 9.92 kWh/FU (Scenario A: 0.057 kWh/kg x 174 kg) and 7.42 kWh/FU (Scenario A: 0.057 kWh/kg x 130 kg). The major emissions to air were produced from the grinding process in the form of mass loss (dust). The study was performed by Surovtseva et al. (2022), who estimated these values as 0.4 % for emissions and as2% fora mass loss in case of synthetic graphite, which had almost similar chemical composition to SR [52]. These values were used as an inventory data for the mechanical process after scaling to FU and the values are presented in **Table [S2]**.

##### 2.3.6.2 Life cycle inventory of thermal treatment

The energy consumed during the thermal treatment (pyrolysis or catalytic pyrolysis) at 600 °C was measured in the laboratory using pyrolysis plant and estimated at 0.15 kWh/kg [27], what means 150 kWh/FU. The amount of N_2_ gas to decompose 1 ton of plastic waste was adopted as 32 kg (900 L) [36]. In case of catalytic pyrolysis, the laboratory experiments confirmed that the catalyst could be regenerated for reuse [6], so the end-of-life scenario of the zeolite catalyst was excluded in the proposed LCA model. Since the current research focuses on MFPW recycling, catalyst production is not included in the calculation, especially since spent catalyst can be regenerated and reused again in the conversion process of MFPW, where the study performed by Ahamed et al. (2020) assumed that a 2 % loss would only occur during the implementation of the recovery step [6]. 5 % were disposed by landfilling. Wax, oil, and gas were handled as an output from this process with higher heating values estimated at 45 MJ/kg, 24 MJ/kg, and 6.7 MJ/kg, respectively [53,54]. Based on these values and yield, the HHV of wax (8820 MJ/FU), oil (5035 MJ/FU), and syngas (4221 MJ/FU “Scenario A″ and 4422 MJ/FU “Scenario B″) were calculated. With regard to solid residue (char or ash), usually it is disposed in landfill [6,55], but in the present research, ash part was neglected from this stage since it will be reprocessed into high added-value products in the following stages. Finally, on-site emission of MFPW was estimated based on values reported by Khoo (2019) [56], thus presenting the output emission of MFPW pyrolysis. **Table [S1]** summarizes the inventory data collected from the literature and lab and Ecoinvent 3.7 database. The pyrolysis process was chosen as a baseline to determine less environment-friendly alternative.

##### 2.3.6.3 Life cycle inventory of chemical treatment

Two chemical processes (leaching and functionalization) were involved in the proposed LCA model for treating SR fraction into AlCl₃ and CPs. Unfortunately, there is a lack of an inventory database for the synthesis of these materials. Even the LCA study conducted by Ahmed et al. (2020) assumed that Al could be recovered and separated from the ash fraction without going into details of the extraction and purification processes [6]. Therefore, the chemical preparation of AlCl₃ and CPs from SR was modelled and compared with preparation of these materials from Al and graphite. The LCA model for these operations was first built based on the data available in the literature sources and then chemical and thermal methods were included into the system since these routes were used commercially for producing of Al salt and CPs. The inventory data of these processes were taken from Surovtseva et al. (2022) results, who prepared both elements by similar methods [52]. Since the leaching process was performed at room temperature, no input power was required for this stage and HCl was considered the only input element. Based on the optimal leaching conditions, a liquid to-solid ratio (L/S: HCl/Al) was 2 l/kg. This means that 176 l of HCl were needed to leach Al (88 kg/FU) for both scenarios in the form of AlCl₃ (418.5 kg/FU), as the amount of Al was constant in both cases. Meanwhile, the energy consumed for the functionalization process at 140 °C for 1 h was adapted experimentally as 0.266 kWh/kg [31]. Besides, 0.44 kWh/kg were consumed during calcination process at 230 °C for 3 h [32]. CPs was refined at L/S of 3 l/kg in an acid mixture composed of 2.25 l of H2SO4 and 0.75 l HNO3 = 3:1. Based on that, 90–184 l/FU of H2SO4 and 30–61 l/FU of HNO3 are needed to purify CPs according to both scenarios. Regarding estimations, most of estimations were assumed as produced from the spent chemicals, including the chemical waste HCl solution, the used H2SO4, and the used HNO3. Since some of these chemicals were consumed during the reactions and some technology for that purpose already exists, these chemicals are neglected. Finally, the chemicals and mass balance for chemical processes and the data for chemicals input to purify CPs and waste output and estimations are shown in **Table [S3]** with help of ‘ecoinvent’ database.

#### 2.3.7 Life cycle inventory analysis

The analysis of the MSR system was performed to address environmental concerns at midpoint stage using the ReCiPe method (Based on midpoint weighting scores approach), where, the analysis was focused on several impact categories (listed in Table [2]), where these categories are classified as most frequently used items in the investigation of waste management and energy recovery systems [57].

**Table [2]:** Results of characterizing environmental impacts of the suggested scenarios.

Abbreviation	Impact category	Unit	**Scenario (A)**	**Scenario (B)**	**Scenario (C)**	**Scenario (D)**	**Scenario (E)**
GW	Global warming	kg CO2 eq	3.29E-01	4.17E-01	4.62E-01	7.66E-01	7.52E-01
SOD	Stratospheric ozone depletion	kg CFC11 eq	4.59E-08	8.90E-08	5.73E-07	5.35E-07	5.33E-07
IR	Ionizing radiation	kBq Co-60 eq	9.25E-04	9.69E-04	1.09E-03	1.76E-03	1.74E-03
OFH	Ozone formation, Human health	kg NOx eq	9.15E-01	9.15E-01	9.15E-01	1.60E+00	1.60E+00
FP	Fine particulate matter formation	kg PM2.5 eq	1.11E+00	1.11E+00	1.11E+00	1.94E+00	1.94E+00
OF-TE	Ozone formation, Terrestrial ecosystems	kg NOx eq	1.47E+00	1.47E+00	1.47E+00	2.58E+00	2.58E+00
TA	Terrestrial acidification	kg SO2 eq	3.81E+00	3.81E+00	3.81E+00	6.68E+00	6.68E+00
H	Freshwater eutrophication	kg P eq	1.17E-05	2.90E-05	4.68E-05	4.19E-05	4.16E-05
ME	Marine eutrophication	kg N eq	1.65E-06	9.75E-05	2.01E-04	1.08E-04	1.08E-04
TE	Terrestrial ecotoxicity	kg 1,4-DCB	6.06E-01	6.50E-01	7.01E-01	1.12E+00	1.11E+00
FWE	Freshwater ecotoxicity	kg 1,4-DCB	1.23E-04	3.00E-04	4.79E-04	4.20E-04	4.18E-04
ME	Marine ecotoxicity	kg 1,4-DCB	5.05E-04	7.68E-04	1.05E-03	1.20E-03	1.19E-03
HCT	Human carcinogenic toxicity	kg 1,4-DCB	1.72E-03	2.71E-03	3.81E-03	4.14E-03	4.07E-03
HNCT	Human non-carcinogenic toxicity	kg 1,4-DCB	2.11E-02	6.14E-02	1.04E-01	8.18E-02	8.15E-02
LU	Land use	m2a crop eq	4.98E-03	5.49E-03	6.13E-03	9.35E-03	9.19E-03
MRS	Mineral resource scarcity	kg Cu eq	1.75E-03	1.98E-03	2.22E-03	3.34E-03	3.33E-03
FR	Fossil resource scarcity	kg oil eq	1.78E-01	1.89E-01	1.97E-01	3.35E-01	3.33E-01
WC	Water consumption	m3	6.07E-03	8.36E-03	2.43E-02	4.92E-03	4.96E-03
S	Total score		8.45E+00	8.63E+00	8.76E+00	1.51E+01	1.51E+01

## 3 Results and discussions

**Figure (4):**
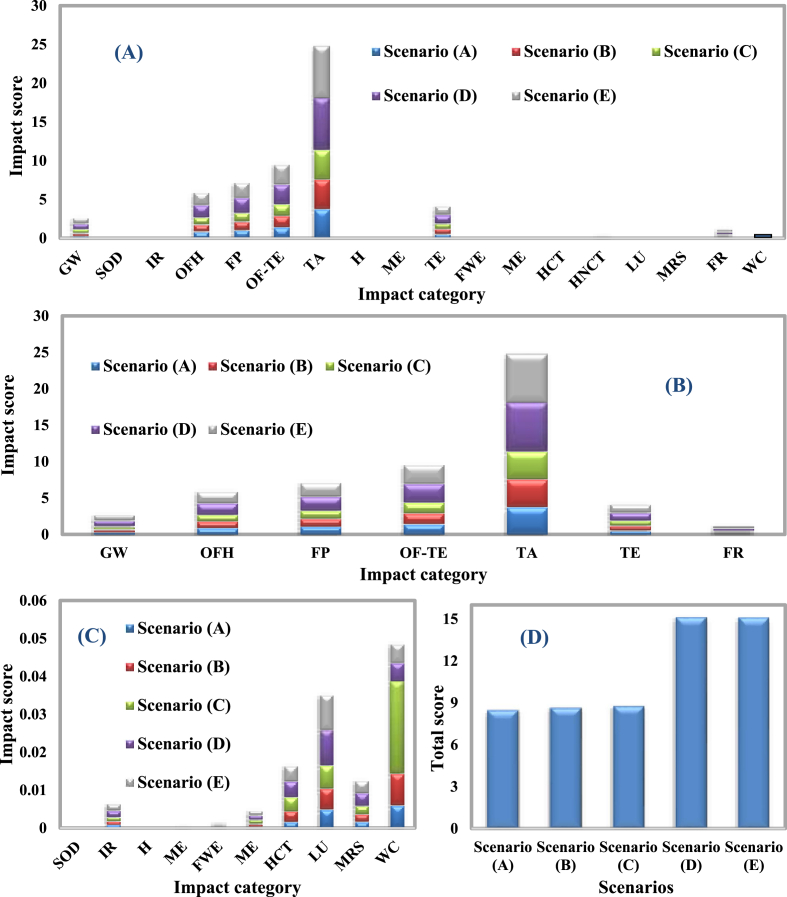
A) distribution of all the environmental impact categories of the developed MSR system, B) distribution of the categories with the biggest effect, C) distribution of moderate categories, and D) the total score of all the suggested scenarios.

The LCA results of MSR system were presented in form of five separate sections distributed according to the environmental impacts of each scenario. The impact score was calculated based on weighting factors. Table [2] shows the environmental impacts of each scenario and its characteristics based on the Midpoint approach. Also, the contributions of these categories were collected and presented together in a single plot based on its unit. as shown in Fig. (4A). In all scenarios, the negative values referred to environmental benefits, while the positive values denoted more environmental burdens. The analysis showed that terrestrial acidification (TA) had the most important effects followed by the ozone formation, terrestrial ecosystems (OF-TE), fine particulate matter formation (FP), ozone formation, human health (OFH), ozone formation, terrestrial ecosystems (OF-TE), and global warming (GW) factors, respectively in all the suggested scenarios. Besides, some moderate impacts of other items had the following trends (from higher to lower): land use (LU), human carcinogenic toxicity (HCT), mineral resource scarcity (MRS), ionizing radiation (IR), marine ecotoxicity (ME), and freshwater ecotoxicity (FWE), respectively. The rest of categories did not manifest any significant effect. Therefore, the analysis process was focused on the most effective categories and the factors with a moderate effect. The credit of these categories and their total score are shown in Fig. (4B-D). As shown in the figures, the influence of the impact of these categories and their total score changed based on the type of treatment and the proposed scenario. All these impacts are described and analysed in the following sections for each scenario.

### 3.1 Environmental impacts of pyrolysis of MFPW

As indicated, the treatment of MFPW via pyrolysis process (baseline scenario) showed some environmental burdens, especially in the most affected categories and these impacts were estimated at GW (0.33 kg CO2 eq), OFH (0.9148 kg NOx eq), FP (1.105 kg PM2.5 eq), OF-TE (1.474 kg NOx eq), TA (3.81 kg SO2 eq), TE (0.61 kg 1,4-DCB), and FR (0.178 kg oil eq). Also, few environmental burdens were observed in the moderate categories, which were estimated at HCT (0.00172 kg 1,4-DCB), LU (0.0049 LU), MRS (0.0017 kg Cu eq), and water consumption (0.0061 m^3^). Most of these burdens resulted from energy consumption during starting up of the pyrolysis plant and leaded to degradation of MFPW [4,27]. Besides, the direct emissions from treatment and generation of large amount of solid waste (17.4 %) needed to be landfilled [36]. However, this scenario showed total score (8.45) higher than that obtained from processing by incineration (6.7–7.8) with 26 % improvement [56,58], due to higher heating value of the recovered wax product (45 MJ/kg) [54]. This demonstrated that the treatment of MFPW via pyrolysis is very effective from the environmental perspective and when compared with incineration.

### 3.2 Environmental impacts of Al leaching

Although the leaching process succeeded in extracting Al fraction, it increased some environmental burdens on the entire system in the GW (27 %), TE (7 %), and FR (6 %) compared to scenario (A). Meanwhile, the other significant factors (OFH, FP, OF-TE, and TA) showed no impacts. Some moderate factors were affected a little and a significant reduction in water consumption (−238 %) was observed. As shown, the hotspots were using HCl for acid leaching process. These results were expected because of acid resulting from production of more chemical residues and production of some dust from the grinding process, in addition to some other factors resulting from losing the extracted Al during washing and filtration processes. All these factors kept the overall score nearly the same when compared to scenario (A). Therefore, it is highly recommended to use some green or bioleaching methods to avoid producing more chemical waste and acid washing [59,60].

### 3.3 Environmental impacts of char functionalization

In this scenario, almost the same manifestations as in the case of leaching treatment were observed with a few additional environmental burdens. The burdens of the most effective categories were increased by GW (40 %), TE (16 %), and FR (11 %), while OFH, FP, OF-TE, and TA factors stayed without any impact. The similar features were noticed in the moderate categories, which were not affected a lot and water consumption decreased by −500 %. This is due to the use of many chemicals in purification of char (coal), resulting in many chemical wastes (spent solutions) and solid wastes resulting from the loss of some elements during purification and filtration [51,52]. Besides, dust was generated during separation and grinding and energy was consumed in the purification and calcination process. All these reasons made the overall outcome of this scenario similar to scenarios (A, B). Therefore, although the coal purification process is promising and economically rewarding, the selection of environmentally friendly methods still requires further studies and chemicals less harmful to the environment need to be selected. H2SO4 and HNO3 were the main hotspots for dust emissions and weight loss during the treatment. However, some environmental burdens were observed in the water consumption category (Fig. (4C)), which increases by adding a new chemical process (e.g., leaching and functionalization) to the LCA boundaries due to the consumption of more water in Al and CPs refining and washing process, which requires equipping this technology with water treatment unit like membranes purification system [61,62].

### 3.4 Environmental impacts of wax upgrading

The results showed that this scenario has a higher overall score of 79 % compared to scenario (A) due to upgrading of the wax product. Despite of that, the catalyst contributed significantly to pyrolysis oil synthesis process (21 %) and decrease of solid residue fraction to (13 %); some additional environmental burdens were estimated at GW (132 %), OFH (133 %), FP (75 %), OF-TE (75 %), TA (75 %), TE (85 %), and FR (89 %) with water consumption reduction estimated at (−181 %). These impacts were caused by burial of the rest of catalysts (2 wt%) and their acidity affecting the soil. Therefore, future research should focus on developing of the approaches of catalyst cleaning where they could be recycled or reused without producing more waste.

### 3.5 Environmental impacts of utilizing of upgraded wax in electricity production

This scenario showed that using oil to produce electricity can reduce the environmental burden compared to scenario (D). Almost the same values were noticed as in the case of scenario (D), where the items were estimated at GW (128 %), OFH (75 %), FP (75 %), OF-TE (75 %), TA (75 %), TE (83 %), and FR (87 %) without big reduction up to 2 %. However, the results were not as desired due to the weak performance (35 %) in electricity production leading to almost the same overall score [63]. Therefore, it is highly recommended to focus on improving the efficiency of electricity production by using the promoted advanced technologies.

## 4 Conclusion

In the present work, the life cycle analysis (LCA) of conversion of metallised food packaging plastics (MFPW) into wax or oil, gas, aluminium chloride (AlCl₃), and carbon microparticles (CPs) via integrated thermal-mechanical-chemical system (MSR) was performed. The analyses were performed based on five scenarios from the perspective of product recovery and its utilization in electricity generation. The results showed that the pyrolysis pathway of MFPW has a better benefits from environmental perspective compared to other scenarios. Meanwhile, the extraction of Al and CPs using leaching and functionalization in addition to the main treatment led to a significant increase in the category of terrestrial acidification, without big changes in the overall score. Also, the wax upgrading scenario (using catalytic pyrolysis) and its applications in electricity production showed a significant increase in the overall score amounting to 79 % with a huge reduction in water consumption. Although the MSR system has a high recycling potential for MFPW, it adds more environmental burdens (e.g., terrestrial acidification), especially leaching and functionalization processes that need to be solved, through using green approaches like bioleaching and enzymatic processes. The use of advanced catalyst regeneration technologies and secondary treatment of chemical waste is also highly recommended to reduce emissions.

## Data availability

The authors confirm that the data supporting the findings of this study are available within the article and its supplementary material.

## CRediT authorship contribution statement

**Samy Yousef:** Writing – review & editing, Writing – original draft, Visualization, Validation, Supervision, Software, Resources, Project administration, Methodology, Investigation, Funding acquisition, Formal analysis, Data curation, Conceptualization. **Inga Stasiulaitiene:** Software, Methodology, Investigation, Formal analysis, Data curation.

## Declaration of competing interest

The authors declare that they have no known competing financial interests or personal relationships that could have appeared to influence the work reported in this paper.
