# Supplementary material for: Life cycle assessment of recycling metallised food packaging plastics using mechanical, thermal and chemical processes
Source: Heliyon. 2024 Aug 19;10(16):e36547. doi: 10.1016/j.heliyon.2024.e36547 (PMC11385764; doi:10.1016/j.heliyon.2024.e36547)
Supplement: Multimedia component 1 [file mmc1.docx]

**Table [S1]:** Inventory data of pyrolysis and catalytic pyrolysis of FU of MFPW

| **Parameter** | **Definition** | **Pyrolysis** | **Catalytic pyrolysis** |
| --- | --- | --- | --- |
| Input | Electricity for pyrolysis | 150 kWh/FU | |
| Outputs | Pyrolysis wax or oil | 8820 MJ/FU | 5035 MJ/FU |
|  | Gaseous product | 4221 MJ/FU | 4422 MJ/FU |
| Emissions to air | CO_2_ | 150 kg/FU | |
|  | CO | 0.07 kg/FU | |
|  | NO_x_ | 0.6 kg/FU | |
|  | SO_2_ | 33600 kg/FU | |
|  | NMVOC | 44800 kg/FU | |
|  | PM (dust) | 95200 kg/FU | |

**Table [S2]:** Inventory data for the mechanical process of FU of MFPW

| Parameter | Definition | Shredding | Grinding | |
| --- | --- | --- | --- | --- |
|  |  |  | Scenario (A) | Scenario (B) |
| Input | Electricity | 15.125 kWh/FU | 9.92 kWh/FU | 7.42 kWh/FU |
| Output | -------------------- | | | |
| Emissions to air | PM (0.4% dust) | ----------- | 0.7 kg/FU | 0.52 kg/FU |

**Table [S3]:** Inventory data for the chemical treatment of SR and ACP

| **Parameter** | **Definition** | **Leaching** | | **Functionalization** | |
| --- | --- | --- | --- | --- | --- |
|  |  | Scenario (A) | Scenario (B) | Scenario (A) | Scenario (B) |
| Inputs | Electricity for calcination | --------- | | 36 kWh/FU | 17.6 kWh/FU |
|  | Electricity for Functionalization |  |  | 21.7 kWh | 10.6 kWh |
|  | HCl | 176 l (289 kg)/FU | | --------- | --------- |
|  | H_2_SO_4_ | --------- | --------- | 184 l (338 kg)/FU | 90 l (166 kg)/FU |
|  | HNO_3_ | --------- | --------- | 61 l (92.3 kg)/FU | 30 l (45.4 kg)/FU |
| Outputs | AlCl₃ (-5% loss) | 418.5 kg/FU | | --------- | --------- |
|  | CPs (-5% loss) | --------- | --------- | 80 kg | 39.2 kg |
| Emissions | HCl solution (25% consumed in the reaction) | 132 l (217 kg) /FU | | --------- | --------- |
|  | H2SO4 (25% consumed and evaporate) | --------- | | 137.7 l (253 kg)/FU | 67.5 l (124 kg)/FU |
|  | HNO3 (25% consumed and evaporate) | --------- | | 46 l (70 kg)/FU | 22.5 l (34 kg)/FU |
